# Effect of plant density on yield and Quality of perilla sprouts

**DOI:** 10.1038/s41598-020-67106-2

**Published:** 2020-06-18

**Authors:** Liuliu Wu, Zhe Deng, Lifan Cao, Li Meng

**Affiliations:** 1 College of Life Science and Technology, Henan Institute of Science and Technology, Xinxiang, 453003 China; 2 Henan Engineering Research Center of Crop Genome Editing, Xinxiang, 453003 China

**Keywords:** Plant development, Plant development, Plant physiology, Plant physiology

## Abstract

Growth and nutraceutical quality of perilla sprouts is strongly dependent on planting density. This study explored the influence of planting density on growth, photosynthetic parameters, antioxidant capacity, main secondary metabolites, soluble sugar and soluble protein contents of ready-to-eat sprouts. Planting at a density of 1450 plants m^−2^ significantly increased yield, improved the activities of antioxidant enzymes SOD and CAT, enhanced the generation of reactive oxygen species, increased the content of total chlorophyll and net photosynthetic rate, and decreased the content of MDA in perilla sprouts. The content of flavonoids, volatile oil, soluble sugar and soluble proteins was highest when the density was 1450 plants m^−2^ compared to other groups. The relative contents of RA and anthocyanin in perilla sprouts reached the maximum value at planting density of 1887 plants m^−2^.

## Introduction

Sprouts are parts of plants that grow from planted seeds or other plant organs. Sprouts are extensively used as short growth cycles, source of nutritious compounds or simple cultivation methods to enhance the nutritional and functional quality of cereals, pseudo cereals, cruciferous vegetables and legumes^1^.

Perilla (*Perilla friesians (L.)* Britt.) is commonly used a traditional vegetable^2^. Perilla sprouts are also used to prepare and synthesize health-promoting products and even drugs^3^.

High planting density has been adopted in sprouts production systems. Planting density improves photosynthesis, influences plant height, architecture and synthesis of chlorophyll. Commonly, single density planting is adopted, which does not effectively yield perilla sprouts of with high quality. Seeds consume a lot of energy and resources during germination. High amount of antioxidant substances are synthesized during germination^4–6^. Studies show that germinating seeds produce high phenolic content and antioxidants^5^. Moreover, other studies found that perilla is rich in proteins, carbohydrate, vitamin, amino acid, flavonoids, phenols, volatile oil and other secondary metabolites, which are beneficial to human health^7,8^. It is therefore important to develop methods of enhancing the nutritional quality of this plant^8^. Callan *et al*.^9^ showed that planting density affects the yield and quality of aromatic herbs. Other researchers reported that planting density modifies the chemical composition of sprouts from seeds^10–13^. So far, few studies have explored the effect of planting density on nutritional quality and antioxidant level of perilla sprouts.

Optimizing the planting density will not only improve sprouts yield and quality, but also reduce input cost by reducing seed rate and fertilizer usage without reducing yield^14^. In China, few studies have reported the commonly used plant densities. In this study, we explored the influence of planting density on growth, photosynthetic parameters, antioxidant capacity, main secondary metabolites, soluble sugar and soluble protein contents of ready-to-eat sprouts. The aim of this experimentation was to test the hypothesis that planting density would affect yield and expression of quality traits in perilla sprouts.

## Materials and Methods

### Experimental design and cultivation

This study was conducted at the experimental station of the Institute of traditional Chinese medicine, Henan institute of science and technology, Xinxiang, China (N35° 18′ 13.71, E113° 55′ 15.05). The cultivation conditions were monitored and recorded from April, 2014 to October, 2018. Perilla sprouts variety, a wild species, was obtained from the mountains of Huixian county, Xinxiang, China through wild domestication. The plants were grown on sandy soil in a greenhouse of 100 m × 50 m dimensions. Five planting densities were used: 575, 1012, 1450, 1887 and 2325 plants m^−2^, referred as T1, T2, T3, T4 and T5 treatments. All treatments were conducted through a random block design with three treatments and three replications.

Seeds were sown on seedbeds after germination on 15th April, 2014 and then transplanted it into the field at the 2nd leaf stage. For each planting density, the same row spacing was applied. Before sowing, Nitrogen was applied as the basal fertilizer. The following types of fertilizers were used: 225 kg N ha^−1^, 100 kg P_2_O_5_ ha^−1^ and 125 kg K_2_O ha^−1^. The growth of plants was carried out in a greenhouse maintained at an air temperature of 25 °C and air humidity of 75%. Sprouts were harvested at the 8th leaf stage. For each planting density, 50 representative plants were selected to perform experiments. Given that nutrient content levels vary between leaves of different plant parts, leaves of the outer, middle and inner parts of each sample were torn and mixed for sampling. The mean value of each index for the 50 selected plants was taken as the quality index for each treatment density. Samples were kept at 4 °C until measurement.

### Agronomic parameters and yield

Fresh plants were harvested and weighed immediately. Diameter of stem were measured with Vernier caliper (instrumental precision, 0.01 mm). Stems with internode of nearly 2 cm were selected for diameter measurement, three replicates were conducted for every stem internode. To measure the leaf length and width, we used the middle section of a leaf blade, where it is longest and widest, five replicate measurements were conducted for each leaf blade. The height of leaves and length of roots were measured using a ruler with a 1 mm scale, measurements were conducted in three replicates for each leaf and root. The yield of perilla sprouts was measured based on number of whole plants per 1 square meter. Fresh yield and dry field were determined. Dry field was measured after plants were heated at 105 °C for 30 min, 85 °C for 48 h to a constant weight. The yield was calculated for at least three plants in each group (n = 3).

### Measurement of pigment content and photosynthetic parameters

Photosynthetic parameters of perilla sprouts were measured from 9:00 a.m to 11:00 a.m in each planting density using 50 replicates at 8th leaf. Measurements were performed for leaves with uniform growth and symmetrical leaves from the top of the third section. Net photosynthetic rate (Pn), transpiration rate (Tr), intercellular CO_2_ concentration (Ci) and stomatal conductance (Gs) were recorded at 1000 micron^−2s−1^ with Li-6400 portable photosynthetic meter. Chlorophyll-a, chlorophyll-b and carotenoid content assays were carried out using LiXT method^15^. About 0.5 g of fresh leaves were ground and Chlorophyll was extracted using acetone and 90% ethyl alcohol (v/v) at 4 °C for 48 h until the leaves turned blanch. Chlorophyll content was calculated based on absorbance reading from UV/visible spectrophotometer (722 G) at 663, 645 and 470 nm.

### Assessment of antioxidant enzyme activities

Antioxidant enzymes were extracted as follows: 0.5 g fresh leaves of perilla sprouts were homogenized in precooled 50 mmol L^−1^ phosphate buffer (pH 7.8) containing 0.1 mmol L-1 EDTA and 2% (w/v) insoluble polyvinyl pyrrolidone. The reaction mixture was centrifuged at 12 000 g for 20 min at 4 °C. The supernatant was collected for enzymatic assays, including superoxide dismutase (SOD), Peroxidase (POD) and Catalase (CAT).

The activity of SOD was determined based on inhibition of the photochemical reduction of nitro blue tolazoline (NBT) as described by Polle^16^. The supernatant solution (50 ml) was mixed with a solution containing 130 mmol methionine, 75 mmol NBT, 100 mmol EDTA, 50 mmol phosphate buffer (pH 7.8). 20 mmol riboflavin was added to initiate the reaction. The reaction mixture was illuminated by fluorescent lamps at 4 000 Lx for 20 min, while the control group was kept in darkness. The absorbance of miscible liquids was read at 560 nm.

The activity of POD was estimated based on the absorbance of guaiacol oxidation at 470 nm^17^. Briefly, the supernatant solution (0.15 ml) was mixed with 100 mmol potassium phosphate buffer (pH 7.0), 20 mmol guaiacol, 10 mmol H_2_O_2_ for 1 min in a reaction solution (3 ml final volume).

A 3 ml reaction mixture containing 50 mmol phosphate buffer (pH 7.0), 15 mmol H_2_O_2_ and 0.15 ml supernatant was prepared, followed by decomposition of hydrogen peroxide and determination of 1 min CAT activity at 240 nm by absorbance^17^.

The content of malondialdehyde (MDA) was estimated as described by Mir^18^. A total of 0.5 g fresh leaves of perilla sprouts were homogenized in precooled 5 ml of 0.5% phenobarbitone acid and centrifuged at 5 000 g for 20 min. The supernatant solution (1.5 mL) was mixed with 2.5 mL of 5% TCA and 0.5% phenobarbitone acid, heated at 100 °C for 30 min and cooled quickly. Then reaction mixture was centrifuged at 5 000 g for 10 min. The absorbance of the supernatant was measured and used to determine the content of MDA based on absorbance at 532 and 600 nm.

### Measurement of contents of main secondary metabolites

Anthocyanins were extracted as described by Long^19^. Briefly, 0.5 g of fresh leaves were homogenized in 90% methyl alcohol and 0.1% HCL (v/v) and then refrigerated at 4 °C for 48 hours. The level of anthocyanins was determined based on absorbance at 535 nm and 657 nm with a UV/visible spectrophotometer (722 G).

The concentration of total flavonoids in perilla sprouts was determined using aluminium chloride colorimetric method as described by Willet^20^. Briefly, flavonoids were extracted from 0.5 g of ground leaves. A solution containing ground leaves was treated with methanol solution and subjected to ultrasonic treatment for 1 h. Next, the solution was filtered to obtain 25 ml. Thereafter, 0.5 mL of this solution was mixed 10% aluminium chloride (0.1 mL), 1 mol/L potassium acetate (0.1 mL) and distilled water (4.3 mL) and incubated at room temperature for 30 min. The absorbance of this mixture was measured at 415 nm using a UV/visible spectrophotometer (722 G). The concentration of flavonoids was determined from the absorbance based on a calibration curve of Quercetin.

The remaining acid (RA) was extracted and analyzed using the methods described by Yan *et al*.^21^ with minor modifications. Dried leaves of perilla sprouts (0.5 g) were ground into powder, and treated twice with 30% ethanol solution (25 mL) under sonication for 10 min, and then centrifuged at 4500 rpm for 5 min. The supernatant was filtered using a 0.2 um membrane to obtain 50 mL volume. The content of RA was determined with high performance liquid chromatography (HPLC, LC - 20AT). The mobile phase consisted of methanol-1% phosphoric acid (39:61) and was run at rate of 1.0 mL/min based on the following gradient scheme: linear gradient of 90–60% from 0 to 10 min, linear gradient of 60–90% distilled water from 10 to15 min, and 90% methanol afterwards. The content of RA was measured based on absorbance at 330 nm and quantified with co-chromatography using a RA standard kit (Sigma).

Essential oil was prepared according to the procedure described by Burits and Bucar^22^. 75 g of perilla sprouts were dried in the shade and ground. The extraction was continued for 3 h, to steam distillation, using a Clevenger apparatus until sufficient oil was collected. Extraction of the aqueous distillate with n-hexane and removal of the solvent from the extract under vacuum yielded the essential oil.

### Measurement of soluble sugar (TSS) and soluble protein (SP) concentration

About 50 g of fresh leaves were used to measure the concentrations of SP and TSS. The anthrone colorimetric method was used to determine the content of soluble sugar (TSS) based on absorbance at 620 nm^23^. The concentration of soluble protein (SP) was estimated based on absorbance at 595 nm using bovine serum albumin as a standard using Bradford assay method^23^.

### Statistical analysis

Sprouts were arranged randomly in climatic chamber with three replicates (n = 3) per treatment. Statistical differences between treatments were analyzed using Duncan’s multiple range tests in SPSS software (version 20.0; SPSS, Inc.,Chicago, IL, USA). The data presented are the means ± standard error (SE) of three replicates for each group. At a probability level of P < 0.05 considered statistically significant. All tables and figures were created using Origin software 2015.

## Results

### Agronomic parameters

**Figure 1:**
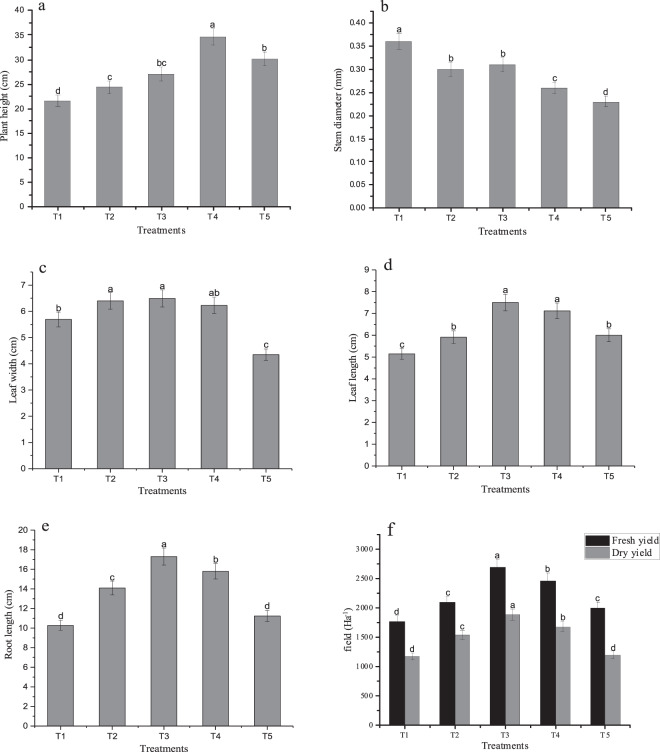
Effects of different planting densities on agronomic characters (**a**–**e**) and yield (**f**) of perilla sprouts Error bars indicate standard error (n = 3). Different the lowercase letters show significance at the 5% level according to Duncan’s test.

Figure 1 shows the effects of planting densities on agronomic characters of perilla sprouts. The height of plants differed between treatments. Notably, the height of plants increased significantly with the increase in planting density. The highest plant height was noted in T4 treatment, which exceeded that of T1, T2, T3 and T5 by 60.37, 42.43, 28.30, and 15.06%, respectively (Fig. 1a). Stem diameter at harvest decreased as the planting density increased. The largest stem diameter was recorded in T1 which reached 0.36 mm (Fig. 1b). This was significantly higher compared to that of T2, T3, T4, and T5 at 20.00, 16.13, 38.46 and 56.52%, respectively. Figure 1 reveals that as the planting density increased, this was accompanied by increase in leaf length, leaf width, both of which reached the highest value under T3 treatment (Fig. 1c,d). Notably, there was no significant difference between T3 and T4 treatment in leaf length. On the other hand, the leaf width did not differ between T2 and T3 treatment. The lengths of root from T3 treatment was 68.45, 22.70, 9.49 and 54.05%, longer than T1, T2, T4 and T5 (Fig. 1e).

Further analysis showed that increase in planting density produced a corresponding increased in fresh yield and dry yield significantly (Fig. 1f). The energetic yield of T3 treatment exceeded that of others treatments by 31.38%. The changes in dry yield at different planting densities followed similar trends as fresh yield. Of note, the dry yield of T3 were observed to be higher as compared to T1, T2, T4, and T5.

### Chlorophyll content and Gas exchange parameters

The content of Chlorophyll (Chl. a, Chl. b, Chl. (a + b), and Chl. a/b) varied at different planting densities. The content of Chlorophyll was higher in T3 treatment than other treatments (Table 1). Samples from T5 treatment group showed the lowest Chl. a, Chl. (a + b) and Chl a/b content of 51.21, 45.1 and 43.47%, respectively, among all treatments. Moreover, the content of Chl. B in T3 treatment was significantly lower by 13.75% compared to T5 treatment, but there was no difference in Chl. B between T1, T2, T4 and T5 treatments.

**Table 1:** Effects of planting density on Chlorophyll content in perilla sprouts.

Chlorophyll (mg g^−1^)	Planting density/(cm/plant^−1^)				
	T1	T2	T3	T4	T5
Chl. a	2.60 ± 0.21d	3.05 ± 0.15c	4.10 ± 0.12a	3.59 ± 0.16b	2.00 ± 0.19e
Chl. b	0.73 ± 0.11b	0.75 ± 0.17b	0.80 ± 0.06a	0.76 ± 0.22b	0.69 ± 0.13b
Chl. (a + b)	3.33 ± 0.05d	3.80 ± 0.08c	4.90 ± 0.04a	4.35 ± 0.08b	2.69 ± 0.12e
Chl a/b	3.56 ± 0.16d	4.07 ± 0.21c	5.13 ± 0.13a	4.72 ± 0.09b	2.90 ± 0.07e

Different lowercase letters denote statistical differences between treatments groups at the 5% level based on Duncan’s test.

Planting densities influenced several photosynthetic parameters of perilla sprouts (Table 2). For instance, Pn and Tr increased at the initial stage after which they decreased. Under T3 treatment, Pn and Tr were 38.21 and 31.80%, respectively, higher than in T1 treatment. The content of Ci was highest in T5 compared to other treatments. In contrast, there were no significant differences in the content of Ci between T3, T4 and T5 treatments. Gs of T4 treatment increased dramatically by 20–50% over other treatments, but no significant differences were found between T1, T2 and T5 treatments.

**Table 2:** Effects of planting density on photosynthetic parameters in perilla sprouts.

Photosynthetic parameters	Planting density/(cm/plant^−1^)				
	T1	T2	T3	T4	T5
*Pn* (umol m^−2^ s^−1^)	8.02 ± 0.11d	11.11 ± 0.08b	12.98 ± 0.70a	10.47 ± 0.13b	8.65 ± 0.50c
*Tr* (mmol m^−2^ s^−1^)	5.94 ± 0.12d	6.77 ± 0.18c	8.71 ± 0.09a	7.45 ± 0.09b	6.73 ± 0.14c
*Ci* (umol m^−2^ mol^−1^)	249.67 ± 0.1b	257.60 ± 0.17b	272.00 ± 0.26a	277.00 ± 0.20a	280.33 ± 0.22a
*Gs* (mol m^−2^ s^−1^)	0.17 ± 0.01c	0.16 ± 0.02c	0.23 ± 0.04b	0.31 ± 0.05a	0.16 ± 0.03c

Different lowercase letters denote statistical differences between treatments groups at the 5% level determined by Duncan’s test.

### Antioxidant enzyme activity

**Figure 2:**
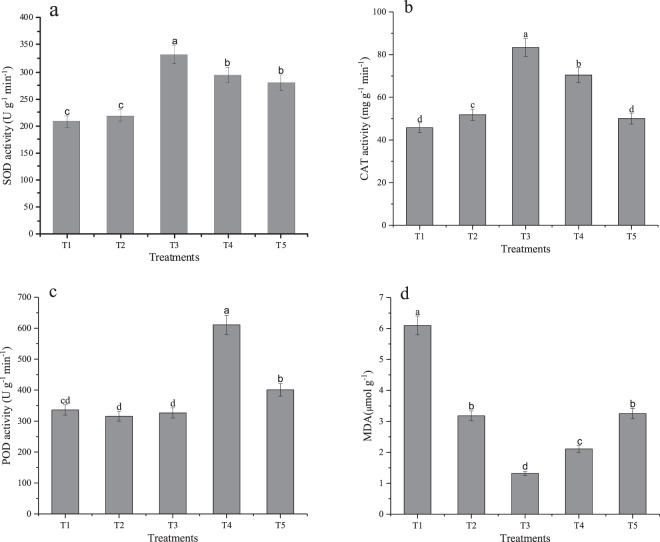
Effects of different planting densities on antioxidant enzyme activity of perilla sprouts (**a**–**d**). Error bars indicate standard d error (n = 3). Different the lowercase letters show significance at the 5% level determined to Duncan’s test.

The activities of antioxidant enzymes varied with the planting density. Initially, the activity of SOD and CAT increased and then decreased with the increase in planting densities. The activity of POD and content of MDA first decreased and then increased with the planting density. The lowest activity of SOD and CAT was recorded in T1 treatment (Fig. 2a,b), while the highest values were recorded in T3 group. Comparatively, the activities of SOD and CAT in T3 group were higher by 59.05 and 82.04%, respectively, than in other groups. The activity of POD was highest under T4, which exceeded that of the lowest value of T2 by 44.98%. However, there was no difference in POD activity between T2 and T3 treatments. Similarly, the content of MDA was highest in T1 group, exceeding that of T2, T3, T4 and T5 groups by 91.8, 362, 189 and 88%, respectively. However, there was no significant difference in MDA contents between T2 and T5 treatments (Fig. 2c,d).

### The main secondary metabolites

Further analysis revealed that the contents of major secondary metabolites in perilla sprouts varied with different planting densities (Table 3). The total flavonoid content under T3 exceeded that of other treatments T1, T2, T4, and T5 by 35.90, 30.28, 15.69 and 55.11%, respectively. Similarly, the content of volatile oil in T3 exceeded that of T1 by 55 and that of T5 by 22.37%. However, there was no difference in the content of volatile oil between T2 and T4 treatments. The content of RA was highest under T4 treatment, which exceeded that of T1 by 24.39%, and that of T5 by 22.68%. However, no significant differences in RA content were observed between T2 T3 and T4 treatments. The content of RA was not significantly different between T1 and T5 treatments. The content of anthocyanins was similar between T3 and T4 groups. There was no significant difference in the content of anthocyanins between T2 and T5.

**Table 3:** Effects of planting densities on the contents of major secondary metabolites in perilla sprouts.

Major secondary metabolites	Planting density/(cm/plant^−1^)				
	T1	T2	T3	T4	T5
Total flavonoid contents (mg.g^−1^)	24.79 ± 0.21 cd	25.86 ± 0.15c	33.69 ± 0.28a	29.12 ± 0.28b	21.72 ± 0.23d
Volatile oil contents (%)	0.60 ± 0.01c	0.89 ± 0.02a	0.93 ± 0.02a	0.90 ± 0.01a	0.76 ± 0.02b
RA contents (mg.g^−1^)	2.87 ± 0.02b	3.37 ± 0.02a	3.50 ± 0.00a	3.57 ± 0.00a	2.91 ± 0.01b
Anthocyanins contents (g^−1^)	6.04 ± 0.09c	7.52 ± 0.00b	8.86 ± 0.08a	8.90 ± 0.03a	7.40 ± 0.01b

Different lowercase letters denote statistical differences between treatments groups at the 5% level determined by Duncan’s test.

### Measurement in TSS and SP concentration

**Figure 3:**
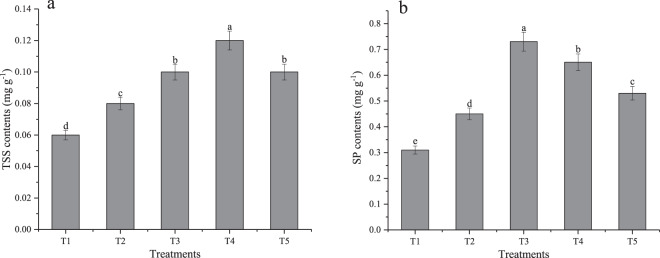
Effects of different planting densities on the contents of TSS and SP in perilla sprouts (**a**–**d**). Error bars indicate standard error (n = 3). Different the lowercase letters show significance at the 5% level determined by Duncan’s test.

Results shown in Fig. 3 indicate that TSS content in T4 group was 100, 50, 20 and 20%, significantly higher than T1, T2, T3, T5. However, the content of TSS was not significantly different between T3 and T5 treatments (Fig. 3a). The content of SP increased with the planting density as shown in Fig. 3b. Of note, the content of SP in T3 treatment exceeded that of T1, T2, T4, and T5 by 135.48, 62.22, 12.31 and 37.74%, respectively.

## Discussion

The effect of planting density on the agronomic characters of perilla sprouts has been demonstrated in this study. The higher the planting density, the smaller the space between plants. This results in longer, thinner, smaller and yellowing of leaves of the sprouts. In addition, high planting density inhibits photosynthesis resulting in rotting of seedlings and lower yield. However, high planting density promotes the growth of roots. In this study, the longest root was recorded in T5 group, while the length, width and diameter of cotyledons were highest under T3 and T4 treatments. The growth of cotyledons was inhibited while the growth of cotyl was enhanced as the seeding density increased. A previous study^24^ reported that the number of plants per square meter is directly proportional to the ratio of crop growth rate. The study also showed that high density ensured maximized light interception, high crop growth rate, and crop biomass, all of which increase the total yield. Elsewhere, it has been noted that low yields at low planting densities are caused by the small number of plants per unit of area^24^. Low yields at low planting densities has also been associated with high plant numbers per unit area^25–27^. This may explain why the highest yield was recorded under T3 treatment. Currently, leaves are the main edible parts of Perilla. Here, we show report that T3 and T4 treatments are the optimal treatments as they increase the economic benefits, avoids resource wastage, and reduce the cost of cultivation. If the density exceeds a certain limit, it deteriorates the lighting and ventilation conditions in the population structure. This weakens the utilization rate of light energy and reduces biological and economic production. High planting density also increases inter-plant water content and atmospheric relative humidity. Our results showed that T3 treatment increased relative humidity and hence this treatment is suitable for perilla sprouts from the transpiration perspective. Farquhar *et al*.^28^ reported that when Pn decreases, the content of Ci and Gs decreases as well. This implies that the decrease in Pn may be due to the reduction in Gs. If Gs decreases and Ci increases, the main reason for the decline in Pn at this time is the decreased photosynthetic capacity of mesophyll cells, which reduces the ability of mesophyll cells to use CO_2_, thus increasing the content of Ci. Under T4 and T5 treatment, Pn was negatively correlated with Gs and Ci levels. nder T1, T2 and T3 treatments, Pn was positively correlated with Gs and Ci. This is consistent with findings derived from summer soybean^29^. Our results show that total chlorophyll content, net photosynthetic rate and intercellular CO_2_ concentration reached the maximum levels under T3 treatment.

Reactive oxygen species (ROS) are often produced during photosynthesis in chloroplasts. Planting density affects the antioxidant system of plants. Under normal circumstances, a balance exists between ROS production and elimination in plants. When this balance is disrupted, ROS accumulates causing damage in cells. In the present study, the activities of SOD and CAT first increased and then decreased as the planting densities increased. Notably, the activities of SOD, POD and CAT were the lowest while the content of MDA was the highest under T1 treatment. Studies indicate that ROS metabolism accelerates membrane lipid peroxidation chain reaction^30–32^. Increase in MDA concentration cause cellular damage, hence metabolic disorders^33^. This implies that the T1 treatment is not a suitable density as it increases the destruction of the antioxidant enzyme protection system, leading to accumulation of MDA and destruction cell membrane. Under T3 and T4 treatments, the activities of SOD, CAT and POD were significantly higher while the content of MDA was lower as compared to T1, T2, and T5. These results indicate that T3 and T4 can sharpen the ability of the anti-oxidation forces during the whole treatment process, stimulate cells to enter a sensitized state, effectively control the excessive concentration of ROS and increase the resistance ability of perilla sprouts.

Secondary metabolites form the main active components of medicinal plants. The biosynthesis and accumulation of these metabolites are influenced by the external environmental conditions. Planting density mainly affects the structure of plant population, increases competition among individuals for light, water and nutrients. These internal environmental conditions dependent on planting density affect plant yield and quality^24^. Secondary metabolites of perilla sprouts include total flavonoids, essential oil, rosemary acid and anthocyanin. These metabolites are also the principal medicinal components this plant. In this study, the content of total flavonoids initially increased and then decreased with the increase of planting densities. The highest total flavonoids content was under T3 treatment. It is reported that plant flavonoids anabolism depends on photosynthesis^34^, which also reflects that T3 treatment is beneficial to the growth and light absorption of perilla sprouts, thus promoting the increase of flavonoids content. Anthocyanin, as a flavonoid compound, which could be for this reason that the contents of anthocyanin and flavonoids of perilla sprouts were both higher under T3 treatment. Our results revealed that the content of RA increased with the increase of planting density, being highest under T4 treatment. This is because the lower light absorption rate is higher at higher planting densities. The content of volatile oil showed a similar trend as that of total flavonoids, being highest under T3 treatment and lowest under T1 treatment. This indicates that density has a positive effect on the content of volatile oils, which is consistent with previous observations^35^.

Planting density modifies the photosynthetic rate and photosynthetic carbon assimilation capacity of different parts of leaves. This is so because planting density influences the nutritional status and light distribution characteristics of plants. SP plays a major role in the growth process of plants and is an important component of plant enzymes. It, therefore, plays an important function in overall metabolism of plants^36^. In this study, we show that planting density influences SP content in the perilla sprouts. Notably, the content of SP was highest under T3 treatment, which gradually decreased. We speculate that with the narrowing of rows between plants at high planting density, the light energy absorbed by PSII antenna pigment dissipates less in the form of heat as more of it is used for photosynthetic electron transfer^37^. Appropriate competition is important for improving SP content and photosynthetic efficiency. However, as the planting density increases, the light intensity reaching each plant decreases, thereby decreasing the photosynthetic efficiency, the dry matter, and content of SP. The content of TSS in plants not only reflects the synthesis of carbohydrates, but also the status of carbohydrates transport. The concentration of carbohydrate also reflects the influence of external environment on plant growth and development^37^. Our results show that the content of TSS in perilla sprouts increased rapidly at first and then gradually decreased with the increase of planting density (Fig. 3a). Notably, the content of TSS under T4 treatment were significantly higher than in other treatments, which is in agreement with results obtained by Zhang *et al*.^38^. In their study, the content of TSS was highest in wheat leaves planted with narrow rows between plants. The changes in content of TSS in our study differs from previous studies in corn plants^39^. It was noted that the content of TSS decreased in various organs of corn when the planting density increased. Increase in the cytoplasmic content of TSS decreased the permeability of plasma membrane and increased the membrane integrity of plasma membrane, which normalized the physiological activities and functions of cells. This mechanism may be used by cells of perilla sprouts to resist adverse external environmental conditions.

## Conclusion

In conclusion, appropriate planting density will lead to high yield and length of perilla sprouts. It also increases the content of biologically active compounds, such as flavonoid, volatile oil, RA, and anthocyanins, TSS and SP. The activities of antioxidant enzymes in perilla sprouts showed a significant power-dependent increase when exposed to appropriate planting density. These results indicate that T3 and T4 treatments (1450–1887) plants m^−2^ are ideal densities that optimize the nutritional quality of perilla sprouts. However, the mechanism by which T3 and T4 densities improve the quality of perilla sprouts should be further studied.
